# Bispecific antibodies and CLEM: an analytical approach to advanced cell imaging for therapeutic strategies

**DOI:** 10.1186/s42649-024-00106-y

**Published:** 2025-01-20

**Authors:** Han-ul Kim, Young Kwan Kim

**Affiliations:** 1 Department of Biochemistry, College of Natural Sciences, Kangwon National University, Chuncheon, 24341 Republic of Korea; 2 Kangwon Center for Systems Imaging, Kangwondaehak-gil, Chuncheon-si, Gangwon-do 24341 Republic of Korea

**Keywords:** Bispecific antibodies, Correlative light and electron microscopy, cell imaging, Biological imaging techniques, Antibody, Molecular diagnosis

## Abstract

The development of bispecific antibodies (BsAbs) represents a significant advancement in therapeutic antibody design, enabling the simultaneous targeting of two different antigens. This dual-targeting capability enhances therapeutic efficacy, particularly in complex diseases like cancer, where tumor heterogeneity presents a significant challenge for traditional treatments. By bridging two distinct pathways, BsAbs can improve specificity and minimize off-target effects, making them invaluable in therapeutic contexts. Integrating advanced imaging techniques, particularly Correlative Light and Electron Microscopy (CLEM), offers a unique opportunity to visualize the dynamic interactions of BsAbs within cellular environments. CLEM combines the strengths of optical and electron microscopy, allowing researchers to observe real-time antibody-antigen interactions at nanoscale resolution. This synergy not only deepens our understanding of BsAbs’ mechanisms of action but also provides critical insights into their spatial distribution, binding kinetics, and functional dynamics in live cells. In this review, the integration of BsAbs and CLEM paves the way for targeted therapeutic strategies, fostering the development of more effective treatments that can adapt to the complexities of disease pathology.

## Introduction

**Fig. 1:**
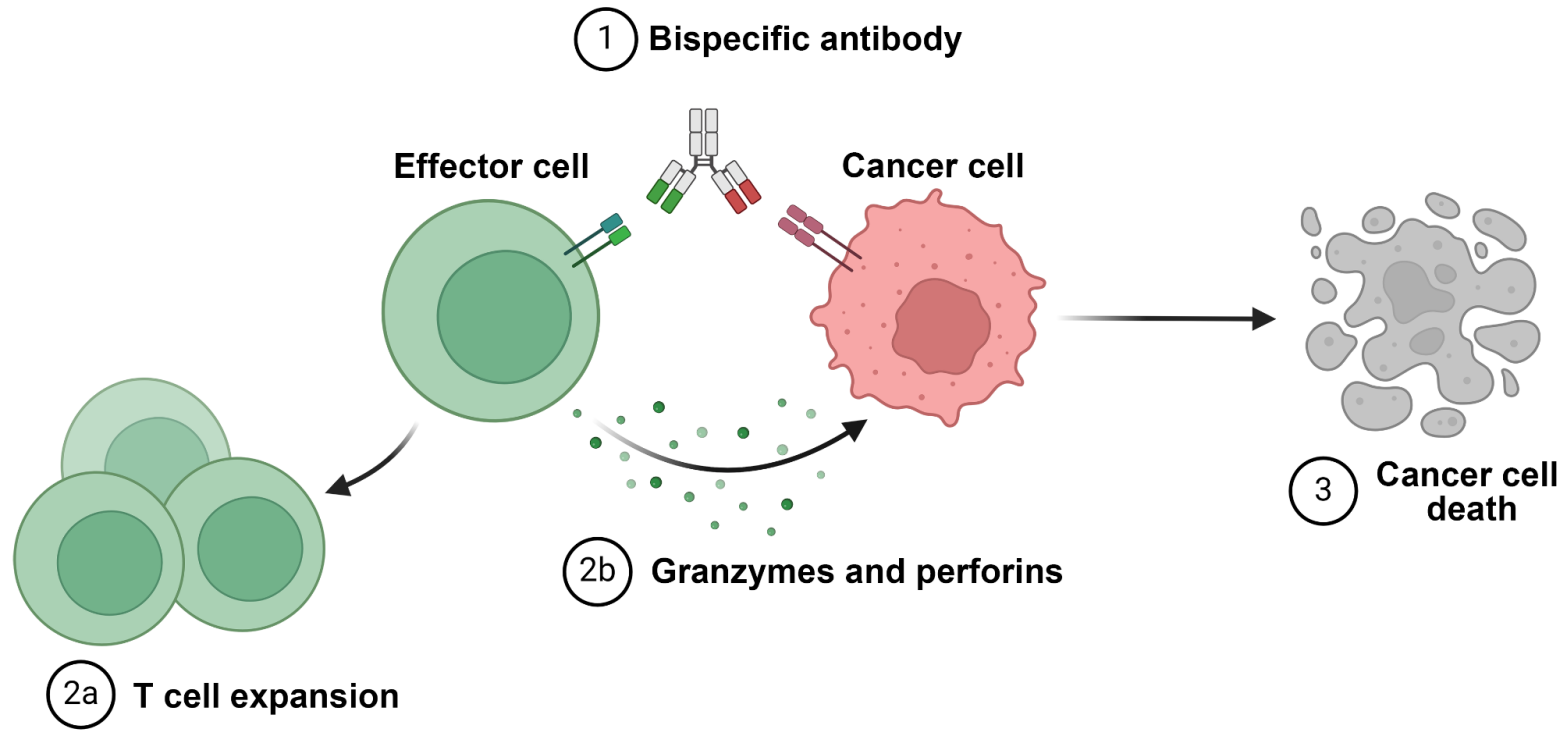
Structure and mechanism of BsAbs. The structural framework of BsAbs is illustrated, highlighting their ability to simultaneously bind to T cells and cancer cells. One end targets a tumor-associated antigen, such as HER2, while the other engages a T cell receptor such as CD3. This dual binding facilitates T cell recruitment to the tumor site, leading to the release of cytotoxic molecules, including granzyme and perforin, which induce apoptosis in cancer cells. *The illustration was adopted from BioRender*

Bispecific antibodies (BsAbs) represent a groundbreaking advancement in the field of immunology and therapeutic development (Khosla et al. 2023; Wei et al. 2022). Unlike traditional monoclonal antibodies that target a single antigen, BsAbs are engineered to bind two different antigens simultaneously (Ma et al. 2021). This dual-targeting capability allows BsAbs to bridge distinct cellular pathways, making them particularly valuable in therapeutic contexts such as cancer treatment, autoimmune diseases, and infectious diseases (Ma 2021; You et al. 2021). The initial concept of BsAbs dates back several decades, but recent advancements in protein engineering have significantly enhanced their development and therapeutic potential (Gong and Wu 2023). Traditional monoclonal antibodies are highly effective in neutralizing single antigens or activating specific immune responses; however, their efficacy can be limited in complex diseases where multiple pathways or antigens are involved (Singh et al. 2023). BsAbs address this limitation by enabling simultaneous engagement with two distinct targets. This not only increases the specificity of the treatment but also enhances its ability to modulate complex biological networks. For example, in cancer therapy, BsAbs can simultaneously bind to a tumor-associated antigen on cancer cells and a T-cell receptor, facilitating the recruitment of immune cells to the tumor site and promoting the direct killing of cancer cells (Trabolsi et al. 2019; van de Donk and Zweegman 2023; Fig. 1). BsAbs such as blinatumomab, which targets both cluster of differentiation 19 (CD19) on B-cell malignancies and cluster of differentiation 3 (CD3) on T-cells, have shown remarkable success in treating certain types of leukemia (Myers et al. 2022; Wudhikarn et al. 2022). Similarly, BsAbs are being developed to target both pro-inflammatory cytokines and immune checkpoint molecules in autoimmune diseases, offering a multifaceted approach to disease management (Nosenko et al. 2017). Furthermore, BsAbs are designed through various approaches, including quadromas, chemical conjugation, and recombinant DNA technology (Liguori et al. 2022). These approaches allow for the precise control of antibody specificity, affinity, and pharmacokinetics, ensuring that the engineered antibodies maintain a high level of effectiveness and stability within the biological system. Advances in genetic engineering have also led to the creation of newer formats, such as tandem single-chain variable fragments (scFvs) and dual-variable domain immunoglobulins (DVD-Ig), which further enhance their therapeutic potential by improving their ability to penetrate tissues and interact with target cells (Khosla 2023; Munoz-Lopez et al. 2022). With this dual functionality, BsAbs hold promise in overcoming resistance mechanisms often seen with traditional monoclonal antibody therapies. The ability to target multiple pathways simultaneously is particularly crucial in diseases where tumor heterogeneity or immune escape mechanisms are common challenges. As research continues to explore new BsAb formats and applications, a deeper understanding of their mechanisms of action is necessary.

This is where advanced imaging technologies, particularly Correlative Light and Electron Microscopy (CLEM), come into play. CLEM integrates the strengths of light microscopy and electron microscopy, allowing researchers to visualize biological processes at both the cellular and molecular levels (Iwasaki et al. 2022; Jun et al. 2019). Light microscopy enables real-time observation of living cells, while electron microscopy provides high-resolution imaging of cellular structures and interactions (Cho et al. 2024). In the context of BsAbs, CLEM is essential for studying their behavior in live cells. It enables researchers to visualize the binding and internalization of BsAbs in real-time, revealing how these antibodies interact with their targets and mediate cellular responses (Kobold 2024; Sun et al. 2023). This dynamic visualization is crucial for understanding the mechanism of action of BsAbs, especially in complex environments like the tumor microenvironment or the immune synapse formation between immune cells and target cells. Moreover, CLEM can elucidate the spatial distribution of BsAbs within cells, allowing researchers to investigate how these antibodies penetrate tissues and reach their intended targets (Thurber et al. 2007). For example, CLEM can be employed to visualize the localization of BsAbs within tumor tissues, providing insights into how effectively they engage with cancer cells (Falchi et al. 2023; Gavriil et al. 2023). The ability to correlate fluorescence imaging of BsAbs with high-resolution structural information from electron microscopy enhances our understanding of the molecular mechanisms that underlie the functionality of these innovative therapeutic agents (Doyle et al. 2024; Liv et al. 2013). The integration of advanced imaging techniques such as CLEM is crucial for unlocking the potential of BsAbs in research and therapeutic applications. As researchers continue to explore the mechanisms of BsAb application, CLEM will play an increasingly vital role in characterizing the interactions of these antibodies with their targets, ultimately paving the way for the development of more effective treatments.

## Main text

### The role of bsAbs in modern therapeutics

BsAbs have emerged as a transformative class of therapeutic agents in oncology, immunology, and beyond (Blanco et al. 2021). These engineered antibodies can bind simultaneously to two different antigens, offering unprecedented opportunities for targeted therapy. This dual-targeting capability enhances their effectiveness compared to traditional monoclonal antibodies, which typically target a single antigen (Ma 2021). One notable application of BsAbs is in cancer immunotherapy, where they target tumor-associated antigens and immune cell receptors. For instance, BsAbs that bind to both CD3 on T cells and human epidermal growth factor receptor 2 (HER2) on breast cancer cells have been developed to redirect T cells to tumor cells, promoting tumor cell destruction (Alaluf et al. 2024; Junttila et al. 2014). This mechanism enhances T cell activation and increases the specificity of the immune response, minimizing collateral damage to healthy tissues. Additionally, BsAbs are being explored for their potential in treating autoimmune diseases and infectious diseases (Zhao 2020). By bridging the gap between pathogenic targets and immune cells, BsAbs can modulate immune responses in a controlled manner, providing new avenues for treatment. Despite their promise, the design and optimization of BsAbs present significant challenges. Understanding their binding kinetics, stability, and pharmacokinetics is crucial for their successful development and application. Advanced imaging techniques, such as CLEM, are instrumental in elucidating the dynamic behavior of BsAbs within biological systems, enabling researchers to visualize their interactions and optimize their therapeutic efficacy.

### CLEM in molecular and cellular imaging

**Fig. 2:**
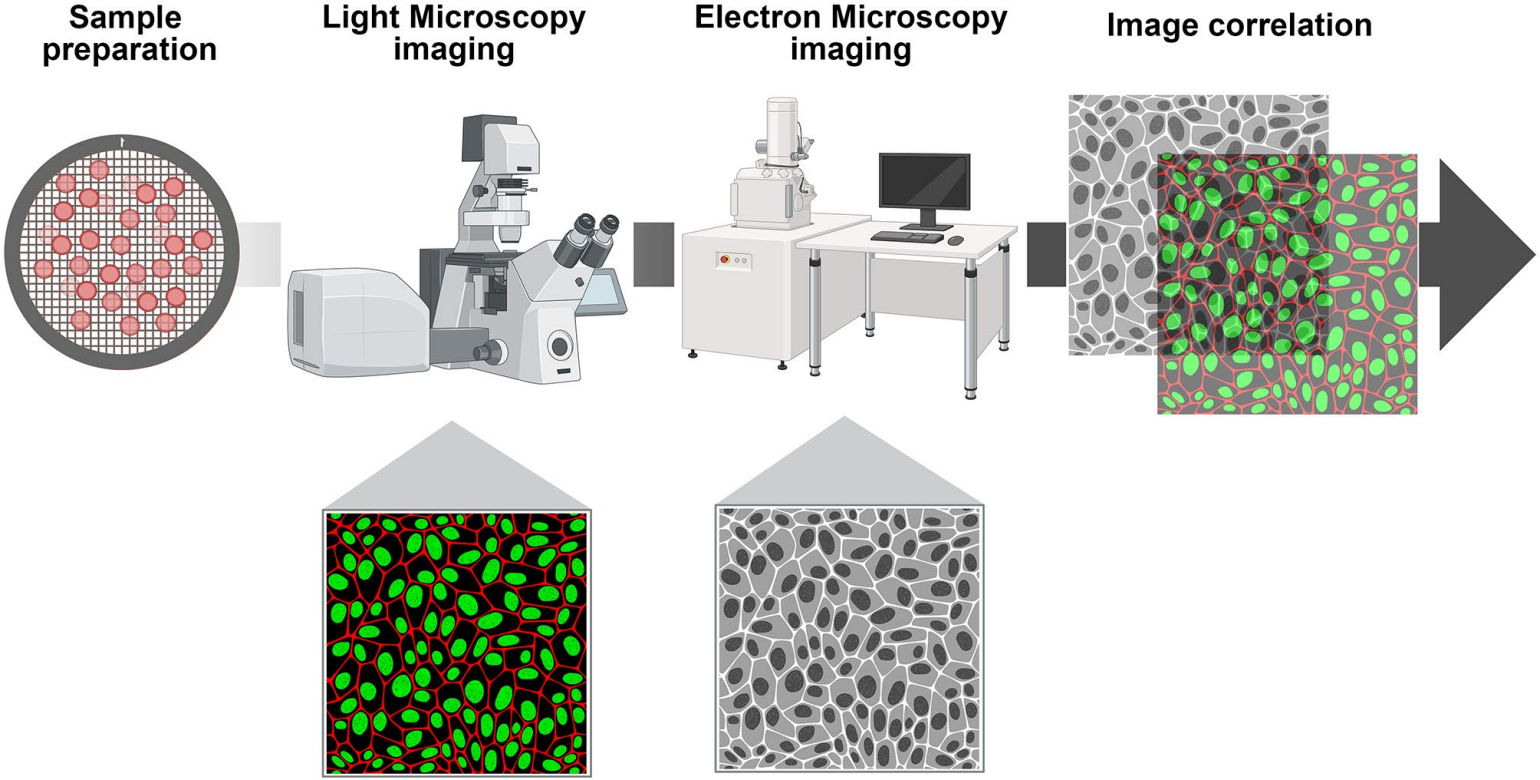
Workflow of CLEM. The schematic diagram outlines the workflow of CLEM. The process begins with sample preparation, where cells are treated with BsAbs. Light microscopy imaging is then performed to visualization of BsAbs with targeted cells, allowing for the tracking of binding and internalization processes. Following this, electron microscopic imaging provides high-resolution structural details of the same samples. The final step correlates the fluorescence imaging data with electron microscopy results, enabling a comprehensive analysis. *The illustration was created in BioRender*

CLEM represents a powerful imaging technique that combines the strengths of fluorescence microscopy and electron microscopy, allowing for the visualization of biological processes at both the cellular and molecular levels (Grabenbauer 2012). It provides a unique opportunity to investigate the dynamic interactions of biomolecules, particularly in the context of BsAbs. Light microscopy allows researchers to observe live cells in real-time, tracking processes such as antibody binding, internalization, and immune synapse formation (Wei 2022). In contrast, electron microscopy provides high-resolution images of cellular structures, revealing intricate details that are often not visible with light microscopy alone. The ability to correlate these two imaging modalities enables a comprehensive understanding of the biological mechanisms at play (Fig. 2). CLEM is essential for studying the behavior of BsAbs in live cells, including visualizing their binding to targets and how they engage with tumor and immune cells. By combining fluorescence imaging of the BsAbs with high-resolution electron microscopy, researchers can gain insights into the molecular interactions and structural changes during antibody-antigen binding (Kobayashi et al. 2016). Moreover, CLEM facilitates the examination of the spatial distribution of BsAbs within tissues, allowing for a better understanding of how these antibodies penetrate and interact with target cells in complex environments. This is particularly important in cancer research, where understanding the localization and dynamics of BsAbs can inform therapeutic strategies.

### Molecular applications of CLEM in BsAbs

The practical applications of CLEM in BsAbs research extend significantly beyond structural characterization, providing critical insights into the behavior and dynamics of these antibodies in live cellular contexts (Tacchetti et al. 2024). One of the key advantages of CLEM is its ability to visualize BsAbs in real-time, capturing dynamic processes as they unfold. This capability is particularly valuable in understanding how BsAbs interact with their targets within the complex architecture of tissues, especially in cancer immunotherapy (Blanco 2021; Wei 2022). CLEM has been effectively utilized to study the spatial dynamics of BsAbs during immune synapse formation. For instance, researchers employed CLEM to observe the behavior of BsAbs designed to target HER2 and CD3. The BsAbs were fluorescently labeled and tracked as they engaged HER2-expressing tumor cells and recruited T cells (Trabolsi 2019). This real-time imaging allowed for the observation of immune cell recruitment dynamics and the subsequent killing of tumor cells. Importantly, the findings were validated through high-resolution imaging techniques, demonstrating the structural characteristics of the immune synapse and highlighting the efficacy of the BsAb in promoting targeted immune responses (Carrasco-Padilla et al. 2022). Furthermore, CLEM is instrumental in studying how BsAbs penetrate tumor tissues and reach their intended targets. By tracking the movement of BsAbs through dense tumor environments using fluorescence, researchers can assess their efficiency in navigating cellular barriers (Thoreau and Chudasama 2022). Another significant application of CLEM in BsAb research is in the investigation of cellular internalization pathways. By correlating fluorescence imaging of BsAbs with electron microscopy, researchers can explore the mechanisms through which BsAbs are internalized by target cells (Cheng et al. 2020). This information is essential for determining the pharmacokinetics of BsAbs and optimizing their therapeutic potential. The ongoing advancements in CLEM techniques are expected to propel the field of BsAb research forward.

### Recent studies in combining BsAbs with CLEM

A significant example of the powerful combination of BsAbs with CLEM can be seen in the research focusing on HER2-positive breast cancer (Zhang et al. 2022). This cancer subtype is characterized by the overexpression of HER2, which is associated with aggressive disease progression and poor clinical outcomes. Researchers have developed BsAbs that target both HER2 on cancer cells and CD3 on T cells, effectively bridging the innate immune system with adaptive immunity. In a pivotal study, researchers utilized CLEM to track the behavior of a specific BsAb designed to bind to HER2 and CD3 (Hu et al. 2024; Lum et al. 2021). Initially, CLEM was employed to visualize the binding kinetics of the BsAb in real-time, allowing researchers to observe how it interacted with HER2-expressing tumor cells. The fluorescence microscopy component of CLEM provided crucial insights into the dynamics of BsAb binding, demonstrating the speed and efficiency with which these antibodies engage their targets. Furthermore, the integration of CLEM into clinical research offers the prospect of personalized therapy. By utilizing patient-derived tumor samples, researchers can evaluate the effectiveness of specific BsAbs in real-time, tailoring treatment strategies to individual patients (Grönholm et al. 2021). This personalized approach enhances the likelihood of successful therapeutic outcomes and contributes to understanding how different tumor microenvironments affect BsAb efficacy. This case study exemplifies the transformative potential of combining bispecific antibodies with advanced imaging techniques like CLEM.

### Advancing analytical approaches: integrating CLEM and cryo-EM for BsAbs

The integration of CLEM with cryo-electron microscopy (cryo-EM) provides an unprecedented opportunity for investigating the dynamics and structural characteristics of BsAbs within complex biological systems (Fernandez-Quintero et al. 2023; Kim and Jung 2021; Li et al. 2020). CLEM allows researchers to visualize real-time interactions between BsAbs and their targets, capturing essential kinetic information about their binding, internalization, and cellular responses (Blanco 2021; Wei 2022). While CLEM excels at tracking these dynamic processes in living cells, cryo-EM offers high-resolution structural insights into the molecular architecture of BsAbs and their interactions at the nanoscale. One of the significant advantages of combining CLEM with cryo-EM lies in the ability to correlate the temporal and spatial dynamics observed during live-cell imaging with detailed structural information acquired through cryo-EM. For example, researchers can utilize CLEM to observe how a BsAb engages with tumor cells and the resultant formation of immune synapses in real-time (Chan et al. 2018; Pillarisetti et al. 2020). Following these observations, cryo-EM can be employed to capture the molecular details of these interactions, revealing the conformational changes that occur during binding. Moreover, the combination of CLEM and cryo-EM enables the study of BsAb behavior in various microenvironments, such as those found in tumors (Xie et al. 2022; Zanetti-Domingues et al. 2024). This integrated approach enhances our understanding of the fundamental principles governing BsAb interactions and aids in optimizing their design for therapeutic applications.

**Fig. 3:**
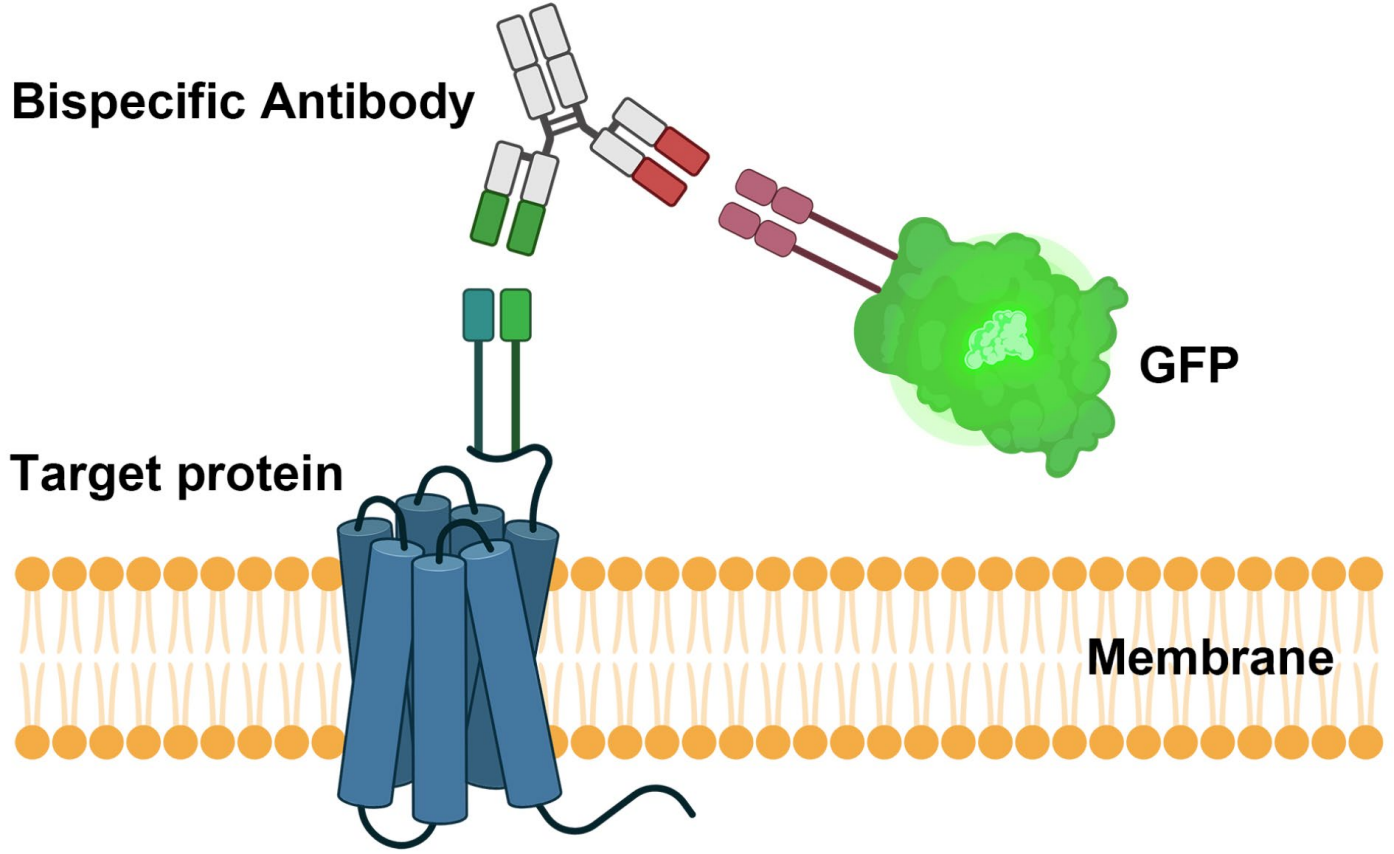
Schematic representation of CLEM applications utilizing BsAbs. Illustration of a BsAbs engineered to bind simultaneously to a target membrane protein and GFP. This strategy enables the visualization and identification of targets on the cell surface. The schematic highlights the role of BsAbs in dual binding to the membrane protein with GFP. *The illustration was created in BioRender*

Furthermore, the application of BsAbs that selectively bind to cell membrane proteins, combined with CLEM, provides a cutting-edge method for elucidating membrane protein interactions at a molecular level. This approach allows for real-time visualization and high-resolution structural assessment, crucial for understanding BsAb interactions with membrane targets such as Green fluorescent protein (GFP)-tagged proteins or tumor-associated antigens. For instance, BsAbs engineered to bind both GFP and specific membrane proteins allow for precise imaging of spatial dynamics on the cellular membrane, a method that facilitates analysis of protein-protein interactions, signaling pathways, and protein distribution within complex cellular environments (Fig. 3). This application is particularly impactful in studying proteins such as HER2, a tumor-associated membrane protein, where BsAbs can bridge HER2 on cancer cells with immune cell receptors, thereby inducing immune synapse formation. Through fluorescence microscopy, CLEM can trace HER2’s membrane distribution and interactions in real time, while electron microscopy provides ultrastructural detail, revealing molecular changes upon BsAb binding that trigger immune cell-mediated cytotoxicity (Pillarisetti 2020). Additionally, CLEM imaging of BsAb interactions with membrane proteins in the tumor microenvironment can unveil dynamic processes such as BsAb internalization, turnover, and their pharmacokinetic behavior. This deepens our understanding of how BsAbs selectively engage with target proteins under physiological conditions, which is essential for optimizing their design and therapeutic potential.

## Conclusion

In summary, CLEM is proving to be a transformative tool in the study of bispecific antibodies. Its capacity to visualize dynamic processes in live cells and correlate these observations with high-resolution structural information opens new avenues for optimizing the design and application of BsAbs in therapeutic contexts. As research progresses, CLEM will undoubtedly continue to play a pivotal role in advancing our understanding of bispecific antibodies and their potential for treating complex diseases. Future research will continue to explore how these imaging techniques can be leveraged to unlock new possibilities for BsAbs, both in the lab and in clinical settings.

## Data Availability

All data generated or analyzed during this study are included in this article and no datasets were generated or analyzed during the current study.

## Abbreviations

BsAbs: Bispecific antibodies
CLEM: Correlative light and electron microscopy
CD3: Cluster of differentiation 3
CD19: Cluster of differentiation 19
scFvs: Single-chain variable fragments
DVD-Ig: Dual-variable domain immunoglobulins
HER2: Human epidermal growth factor receptor 2
Cryo-EM: Cryo-electron microscopy
GFP: Green fluorescent protein

## References

[CR1] E. Alaluf, M.M. Shalamov, A. Sonnenblick, Update on current and new potential immunotherapies in breast cancer, from bench to bedside. Front. Immunol. **15**1287824 (2024) 10.3389/fimmu.2024.1287824 PMC10905744 38433837

[CR2] B. Blanco, C. Dominguez-Alonso, L. Alvarez-Vallina, Bispecific Immunomodulatory antibodies for Cancer Immunotherapy. Clin. Cancer Res. **27**(20), 5457–5464 (2021) 34108185 10.1158/1078-0432.CCR-20-3770 PMC9306338

[CR3] C. Carrasco-Padilla, A. Hernaiz-Esteban, L. Alvarez-Vallina, O. Aguilar-Sopena, P. Roda-Navarro, Bispecific Antibody Format and the Organization of Immunological Synapses in T Cell-redirecting strategies for Cancer Immunotherapy. Pharmaceutics **15**(1), (2022) 10.3390/pharmaceutics15010132 PMC9863865 36678761

[CR4] W.K. Chan, S. Kang, Y. Youssef, E.N. Glankler, E.R. Barrett, A.M. Carter, E.H. Ahmed, A. Prasad, L. Chen, J. Zhang, D.M. Jr. Benson, M.A. Caligiuri, J. Yu, A CS1-NKG2D bispecific antibody collectively activates Cytolytic Immune cells against multiple myeloma. Cancer Immunol. Res. **6**(7), 776–787 (2018) 29769244 10.1158/2326-6066.CIR-17-0649 PMC6030494

[CR5] J. Cheng, M. Liang, M.F. Carvalho, N. Tigue, R. Faggioni, L.K. Roskos, I. Vainshtein, Molecular mechanism of HER2 Rapid internalization and redirected trafficking Induced by Anti-HER2 biparatopic antibody. Antibodies (Basel) **9**(3), (2020) 10.3390/antib9030049 PMC7551206 32961882

[CR6] H.S. Cho, Y.H. Park, S. Moon, C. Park, H.S. Jung, S. Namkoong, Targeting the NTF2-like domain of G3BP1: novel modulators of intracellular granule dynamics. Biochem. Biophys. Res. Commun. **697**, 149497 (2024) 38262290 10.1016/j.bbrc.2024.149497

[CR7] N. Doyle, J. Simpson, P.C. Hawes, H.J. Maier, A novel optimized pre-embedding antibody-labelling correlative light electron microscopy technique. Access. Microbiol. **6**(2), (2024) 10.1099/acmi.0.000750.v3 PMC10928386 38482358

[CR8] L. Falchi, S.A. Vardhana, G.A. Salles, Bispecific antibodies for the treatment of B-cell lymphoma: promises, unknowns, and opportunities. Blood. **141**(5), 467–480 (2023) 36322929 10.1182/blood.2021011994 PMC9936308

[CR9] M.L. Fernandez-Quintero, N.D. Pomarici, A.M. Fischer, V.J. Hoerschinger, K.B. Kroell, J.R. Riccabona, A.S. Kamenik, J.R. Loeffler, J.A. Ferguson, H.R. Perrett, K.R. Liedl, J. Han, A.B. Ward, Structure and Dynamics Guiding design of antibody therapeutics and vaccines. Antibodies (Basel) **12**(4), (2023) 10.3390/antib12040067 PMC10594513 37873864

[CR10] V. Gavriil, A. Ferraro, A.C. Cefalas, Z. Kollia, F. Pepe, U. Malapelle, C. De Luca, G. Troncone, E. Sarantopoulou, Nanoscale prognosis of Colorectal Cancer Metastasis from AFM Image Processing of histological sections. Cancers (Basel) **15**(4), (2023) 10.3390/cancers15041220 PMC9953928 36831563

[CR11] S. Gong, C. Wu, Efficient production of bispecific antibodies-optimization of transfection strategy leads to high-level stable cell line generation of a Fabs-in-tandem immunoglobin. Antib. Ther. **6**(3), 170–179 (2023) 37492586 10.1093/abt/tbad013 PMC10365153

[CR12] M. Grabenbauer, Correlative light and electron microscopy of GFP. Methods Cell. Biol. **111**, 117–138 (2012) 22857926 10.1016/B978-0-12-416026-2.00007-8

[CR13] M. Grönholm, M. Feodoroff, G. Antignani, B. Martins, F. Hamdan, V. Cerullo, Patient-derived Organoids for Precision Cancer Immunotherapy. Cancer Res. **81**(12), 3149–3155 (2021) 33687948 10.1158/0008-5472.CAN-20-4026 PMC7616950

[CR14] L. Hu, S. Zhang, J. Sienkiewicz, H. Zhou, R. Berahovich, J. Sun, M. Li, A. Ocampo, X. Liu, Y. Huang, H. Harto, S. Xu, V. Golubovskaya, L. Wu, HER2-CD3-Fc bispecific antibody-encoding mRNA delivered by lipid nanoparticles suppresses HER2-Positive Tumor Growth. Vaccines (Basel) **12**(7), (2024) 10.3390/vaccines12070808 PMC11281407 39066446

[CR15] H. Iwasaki, S. Ichinose, Y. Tajika, T. Murakami, Recent technological advances in correlative light and electron microscopy for the comprehensive analysis of neural circuits. Front. Neuroanat. **16**(1061078 (2022) 10.3389/fnana.2022.1061078 PMC9748091 36530521

[CR16] S. Jun, H.J. Ro, A. Bharda, S.I. Kim, D. Jeoung, H.S. Jung, Advances in Cryo-Correlative Light and Electron Microscopy: applications for studying Molecular and Cellular events. Protein J. **38**(6), 609–615 (2019) 31396855 10.1007/s10930-019-09856-1

[CR17] T.T. Junttila, J. Li, J. Johnston, M. Hristopoulos, R. Clark, D. Ellerman, B.E. Wang, Y. Li, M. Mathieu, G. Li, J. Young, E. Luis, G. Lewis Phillips, E. Stefanich, C. Spiess, A. Polson, B. Irving, J.M. Scheer, M.R. Junttila, M.S. Dennis, R. Kelley, K. Totpal, A. Ebens, Antitumor efficacy of a bispecific antibody that targets HER2 and activates T cells. Cancer Res. **74**(19), 5561–5571 (2014) 25228655 10.1158/0008-5472.CAN-13-3622-T

[CR18] A.A. Khosla, K. Jatwani, R. Singh, A. Reddy, I. Jaiyesimi, A. Desai, Bispecific Antibodies in Lung Cancer: a state-of-the-art review. Pharmaceuticals (Basel) **16**(10), (2023) 10.3390/ph16101461 PMC10609957 37895932

[CR19] H.U. Kim, H.S. Jung, Cryo-EM as a powerful tool for drug discovery: recent structural based studies of SARS-CoV-2. Appl. Microsc. **51**(1), 13 (2021) 34562174 10.1186/s42649-021-00062-x PMC8464538

[CR20] S. Kobayashi, M. Iwamoto, T. Haraguchi, Live correlative light-electron microscopy to observe molecular dynamics in high resolution. Microscopy (Oxf). **65**(4), 296–308 (2016) 27385786 10.1093/jmicro/dfw024

[CR21] S. Kobold, Giving T-cell bispecifics a helping hand. Blood. **143**(21), 2115–2116 (2024) 38780922 10.1182/blood.2024024346

[CR22] N. Li, Z. Li, Y. Fu, S. Cao, Cryo-EM studies of virus-antibody Immune complexes. Virol. Sin. **35**(1), 1–13 (2020) 31916022 10.1007/s12250-019-00190-5 PMC7035235

[CR23] L. Liguori, G. Polcaro, A. Nigro, V. Conti, C. Sellitto, F. Perri, A. Ottaiano, M. Cascella, P. Zeppa, A. Caputo, S. Pepe, F. Sabbatino, Bispecific antibodies: a Novel Approach for the treatment of solid tumors. Pharmaceutics **14**(11), (2022) 10.3390/pharmaceutics14112442 PMC9694302 36432631

[CR24] N. Liv, A.C. Zonnevylle, A.C. Narvaez, A.P. Effting, P.W. Voorneveld, M.S. Lucas, J.C. Hardwick, R.A. Wepf, P. Kruit, J.P. Hoogenboom, Simultaneous correlative scanning electron and high-NA fluorescence microscopy. PLoS One **8**(2), e55707 (2013) 10.1371/journal.pone.0055707 PMC3568124 23409024

[CR25] L.G. Lum, Z. Al-Kadhimi, A. Deol, V. Kondadasula, D. Schalk, E. Tomashewski, P. Steele, K. Fields, M. Giroux, Q. Liu, L. Flaherty, M. Simon, A. Thakur, Phase II clinical trial using anti-CD3 x anti-HER2 bispecific antibody armed activated T cells (HER2 BATs) consolidation therapy for HER2 negative (0–2+) metastatic breast cancer. J. Immunother Cancer **9**(6), (2021) 10.1136/jitc-2020-002194 PMC8202097 34117114

[CR26] J. Ma, Y. Mo, M. Tang, J. Shen, Y. Qi, W. Zhao, Y. Huang, Y. Xu, C. Qian, Bispecific Antibodies: From Research to Clinical Application. Front Immunol. **12**626616 (2021) 10.3389/fimmu.2021.626616 PMC8131538 34025638

[CR27] P. Munoz-Lopez, R.M. Ribas-Aparicio, E.I. Becerra-Baez, K. Fraga-Perez, L.F. Flores-Martinez, A.A. Mateos-Chavez, R. Luria-Perez, Single-chain Fragment Variable: recent progress in Cancer diagnosis and therapy. Cancers (Basel) **14**(17), (2022) 10.3390/cancers14174206 PMC9455005 36077739

[CR28] R.M. Myers, A. Taraseviciute, S.M. Steinberg, A.J. Lamble, J. Sheppard, B. Yates, A.E. Kovach, B. Wood, M.J. Borowitz, M. Stetler-Stevenson, C.M. Yuan, V. Pillai, T. Foley, P. Chung, L. Chen, D.W. Lee, C. Annesley, A. DiNofia, S.A. Grupp, S. John, D. Bhojwani, P.A. Brown, T.W. Laetsch, L. Gore, R.A. Gardner, S.R. Rheingold, M.A. Pulsipher, N.N. Shah, Blinatumomab Nonresponse and High-Disease Burden are Associated with Inferior outcomes after CD19-CAR for B-ALL. J. Clin. Oncol. **40**(9), 932–944 (2022) 34767461 10.1200/JCO.21.01405 PMC8937010

[CR29] M.A. Nosenko, K.N. Atretkhany, V.V. Mokhonov, G.A. Efimov, A.A. Kruglov, S.V. Tillib, M.S. Drutskaya, S.A. Nedospasov, VHH-Based bispecific antibodies targeting cytokine production. Front. Immunol. **8**, 1073 (2017) 28919896 10.3389/fimmu.2017.01073 PMC5585155

[CR30] K. Pillarisetti, G. Powers, L. Luistro, A. Babich, E. Baldwin, Y. Li, X. Zhang, M. Mendonca, N. Majewski, R. Nanjunda, D. Chin, K. Packman, Y. Elsayed, R. Attar, F. Gaudet, Teclistamab is an active T cell-redirecting bispecific antibody against B-cell maturation antigen for multiple myeloma. Blood Adv. **4**(18), 4538–4549 (2020) 32956453 10.1182/bloodadvances.2020002393 PMC7509877

[CR31] R. Singh, P. Chandley, S. Rohatgi, Recent advances in the development of monoclonal antibodies and next-generation antibodies. Immunohorizons. **7**(12), 886–897 (2023) 38149884 10.4049/immunohorizons.2300102 PMC10759153

[CR32] Y. Sun, X. Yu, X. Wang, K. Yuan, G. Wang, L. Hu, G. Zhang, W. Pei, L. Wang, C. Sun, P. Yang, Bispecific antibodies in cancer therapy: target selection and regulatory requirements. Acta Pharm. Sin B **13**(9), 3583–3597 (2023) 37719370 10.1016/j.apsb.2023.05.023 PMC10501874

[CR33] P. Tacchetti, S. Barbato, K. Mancuso, E. Zamagni, M. Cavo, Bispecific Antibodies for the management of Relapsed/Refractory multiple myeloma. Cancers (Basel) **16**(13), (2024) 10.3390/cancers16132337 PMC11240369 39001399

[CR34] F. Thoreau, V. Chudasama, Enabling the next steps in cancer immunotherapy: from antibody-based bispecifics to multispecifics, with an evolving role for bioconjugation chemistry. RSC Chem. Biol. **3**(2), 140–169 (2022) 35360884 10.1039/d1cb00082a PMC8826860

[CR35] G.M. Thurber, S.C. Zajic, K.D. Wittrup, Theoretic criteria for antibody penetration into Solid Tumors and micrometastases. J. Nucl. Med. **48**(6), 995–999 (2007) 17504872 10.2967/jnumed.106.037069

[CR36] A. Trabolsi, A. Arumov, J.H. Schatz, Cell-activating Bispecific antibodies in Cancer Therapy. J. Immunol. **203**(3), 585–592 (2019) 31332079 10.4049/jimmunol.1900496

[CR37] N. van de Donk, S. Zweegman, T-cell-engaging bispecific antibodies in cancer. Lancet. **402**(10396), 142–158 (2023) 37271153 10.1016/S0140-6736(23)00521-4

[CR38] J. Wei, Y. Yang, G. Wang, M. Liu, Current landscape and future directions of bispecific antibodies in cancer immunotherapy. Front. Immunol. **13**, 1035276 (2022) 36389699 10.3389/fimmu.2022.1035276 PMC9650279

[CR39] K. Wudhikarn, A.C. King, M.B. Geyer, M. Roshal, Y. Bernal, B. Gyurkocza, M.A. Perales, J.H. Park, Outcomes of relapsed B-cell acute lymphoblastic leukemia after sequential treatment with blinatumomab and inotuzumab. Blood Adv. **6**(5), 1432–1443 (2022) 35042232 10.1182/bloodadvances.2021005978 PMC8905691

[CR40] Y. Xie, F. Xie, X. Zhou, L. Zhang, B. Yang, J. Huang, F. Wang, H. Yan, L. Zeng, L. Zhang, F. Zhou, Microbiota in tumors: from understanding to application. Adv. Sci. (Weinh) **9**(21), e2200470 (2022) 10.1002/advs.202200470 PMC9313476 35603968

[CR41] G. You, J. Won, Y. Lee, D. Moon, Y. Park, S.H. Lee, S.W. Lee, Bispecific antibodies: a Smart Arsenal for Cancer immunotherapies. Vaccines (Basel) **9**(7), (2021) 10.3390/vaccines9070724 PMC8310217 34358141

[CR42] L.C. Zanetti-Domingues, M. Hirsch, L. Wang, T.A. Eastwood, K. Baker, D.P. Mulvihill, S. Radford, J. Horne, P. White, B. Bateman, Chapter Eleven - toward quantitative super-resolution methods for cryo-CLEM, in *Methods in Cell Biology*, ed. by T. Müller-Reichert, P. Verkade **187**(Academic, 2024), 249–292. 10.1016/bs.mcb.2024.02.028 10.1016/bs.mcb.2024.02.028 38705627

[CR43] J. Zhang, D. Ji, L. Cai, H. Yao, M. Yan, X. Wang, W. Shen, Y. Du, H. Pang, X. Lai, H. Zeng, J. Huang, Y. Sun, X. Peng, J. Xu, J. Yang, F. Yang, T. Xu, X. Hu, First-in-human HER2-targeted bispecific antibody KN026 for the treatment of patients with HER2-positive metastatic breast Cancer: results from a phase I study. Clin. Cancer Res. **28**(4), 618–628 (2022) 34844975 10.1158/1078-0432.CCR-21-2827

[CR44] Q. Zhao, Bispecific Antibodies for Autoimmune and Inflammatory Diseases: Clinical Progress to Date. BioDrugs. **34**(2), 111–119 (2020) 10.1007/s40259-019-00400-2 31916225

